# Orbitofrontal dysfunction predicts poor prognosis in chronic migraine with medication overuse

**DOI:** 10.1007/s10194-011-0340-6

**Published:** 2011-04-17

**Authors:** Marian Gómez-Beldarrain, María Carrasco, Amaia Bilbao, Juan C. García-Moncó

**Affiliations:** 1 Service of Neurology, Hospital de Galdacano, 48960 Galdacano, Vizcaya Spain; 2 Fundación Vasca de Innovación e Investigación Sanitarias (BIOEF), 48150 Sondika, Vizcaya Spain

**Keywords:** Chronic migraine, Medication overuse, Episodic migraine, Orbitofrontal cortex, Dorsolateral cortex

## Abstract

Chronic migraine patients are at risk of developing a medication overuse. Brain functional studies in these patients have demonstrated an orbitofrontal hypometabolism, persistent after overuse cessation. Orbitofrontal dysfunction is also present in addiction and thus could predispose migraineurs to medication overuse. The aim of this study was to investigate if orbitofrontal dysfunction can be demonstrated in patients with chronic migraine and medication overuse by performing a systematic neuropsychological evaluation focused on tests that assess frontal lobe function. Second, to establish whether it is related to the outcome of these patients. We prospectively studied 42 chronic migraine patients with medication overuse, 42 episodic migraineurs and 41 controls on a battery of neuropsychological tasks evaluating the orbitofrontal and dorsolateral functioning. Depression, anxiety, and personality traits were also assessed. Chronic migraineurs with medication overuse showed a significant impairment in orbitofrontal task performance and higher depression scores as compared to episodic migraineurs and controls. Dorsolateral dysfunction was present in both groups of migraneurs, who also had higher rates of anxiety as compared to controls. After 1 year of follow-up, migraine patient’s outcome was classified according to their medication overuse status. A negative outcome that included persistent or new-onset medication overuse was present in 34% of migraineurs and was associated with baseline poor orbitofrontal task performance, and with mild dorsolateral dysfunction, higher rates of depression, anxiety and neuroticism-anxiety traits. Formal education and years with migraine did not influence outcome. Orbitofrontal dysfunction is present in patients with chronic migraine and medication overuse, and associates with a poor outcome at 1 year of follow-up. Neuropsychological evaluation in migraine may help to detect patients prone to overuse so that appropriate therapeutic attitudes can be taken.

**Electronic supplementary material:**

The online version of this article (doi:10.1007/s10194-011-0340-6) contains supplementary material, which is available to authorized users.

## Introduction

Medication overuse occurs predominantly in patients with a primary headache disorder, particularly migraine patients with a high attack rate [1–4]. Approximately, 10% of migraine patients develop chronic migraine (CM), a condition characterized by an insidious increase of headache frequency and intensity that often results in medication overuse and deterioration of the patient’ quality of life, also representing a significant economic burden [5].

The pathophysiology of this challenging condition is unclear, but several lines of evidence suggest that neuropsychological mechanisms, particularly orbitofrontal (OF) cortex dysfunction, are involved. A PET study that used 18-FDG in 16 chronic migraineurs with medication overuse revealed a persistent OF hypofunction before and 3 weeks after medication withdrawal as compared to healthy controls [6].

OF hypofunction impairs reward and decision-making mechanisms [7, 8], occurs in substance abusers, and could contribute to medication overuse in CM patients. Dorsolateral (DL) executive dysfunctions, including planning, problem solving, and set-shifting tasks have also been described in patients with migraine [9, 10], although not clearly related to medication overuse.

Taken together, these findings suggest that medication overuse might be a consequence of a susceptibility to substance abuse due to prefrontal, particularly OF, dysfunction [6].

In this study, we sought to investigate if OF dysfunction can be demonstrated by performing a systematic neuropsychological evaluation focused on tests that assess prefrontal lobe functions. We also tried to determine if there is a relationship between OF functioning and the outcome of migraine patients in terms of medication overuse.

To this end, we compared a group of chronic migraineurs with medication overuse with episodic migraineurs and with controls, and followed them for 1 year.

## Design and methods

### Subjects

Three groups of individuals were included in this study:

- Forty-two patients suffering from chronic migraine and medication overuse (chronic migraine group—CM group), were consecutively recruited from the Neurology department at our institution. Inclusion criteria were age 55 or younger suffering from chronic migraine and medication overuse according to the current criteria of the International Headache Society [3, 11], i.e., definite or probable migraine headaches present on >15 days/month for >3 months and with an intake of simple analgesics on ≥15 days per month or of any combination of ergotamine, triptans, analgesics, and/or opioids on ≥10 days per month on a regular basis for >3 months. Exclusion criteria included a past history of any neurologic disease different from migraine or a past history of any psychiatric disorder, except for depression or anxiety. We also excluded patients with other chronic pain conditions or under medication for general medical diseases or taking psychotropic drugs.
- Forty-two consecutive patients with episodic migraine without aura [11] (episodic migraine group—EM group).The same exclusion criteria as for the CM group were applied.
- Forty-one healthy subjects (control group—CG group), specifically without headache disorder or any other neurological or psychiatric disease, and under no psychoactive therapy.

All participants had a normal neurologic examination. All migraineurs had a normal brain MRI or CT scan. Only six chronic migraine patients and four patients with episodic migraine had small white matter hyperintensities consistent with those described in patients with migraine. After enrolment, they kept a headache calendar to register the number of days with headache and to keep a medication record.

Instructions on how to manage a migraine attack with NSAIDs and triptans were given to all patients, and appropriate preventive medication was selected or modified as needed on an individual basis. We avoided using opioids in these patients. Patients in the CM group were advised to cease medication overuse and were managed in an outpatient setting.

Patients (CM and EM) were followed for 1 year with scheduled visits at 3-month intervals. Special emphasis was placed in evaluating the status with regards to medication overuse (i.e., whether overusers converted to a non-overuser state and vice versa).

At the end of the follow-up, we classified migraine patients in two groups in terms of medication overuse: those with a positive outcome (EM patients that remained episodic and without medication overuse, and CM overusing medication that ceased overuse) and those with a negative outcome (overusers that persisted on this status after 1 year of follow-up, and EM patients that started overusing). We analyzed the differences in the baseline task performance between both groups and whether the outcome was related with baseline prefrontal functioning.

The study was approved by the Ethics Committee of our Institution and all participants signed a written consent according to the Helsinki recommendations.

### Neuropsychological evaluation

During examination, headache was assessed by a visual analogue scale ranging from zero (no pain) to ten (worst headache); patients scoring ≥4 were asked to perform the evaluation at some other time. The examination was carried out in two sessions of 1 h duration.

#### Orbitofrontal assessment

The three following tasks, that have been shown to be sensitive to ventromedial and OF damage, were employed [12–17].

1. “Reading the Mind in the Eyes” This task consists of photographs of the eye region of 36 different faces. The subject is required to make a choice between four words printed on top and bottom of the picture, and choose the one that best describes the feeling or thought of the subject in the photograph.
2. “Faux-Pas” In this test, the subject is read ten stories that contain a social Faux-Pas and ten stories describing a minor conflict, but in which no Faux-Pas is committed. The text of each story is placed in front of the subject so as to reduce the demands on working memory. The subject should identify the Faux-Pas stories. If a Faux-Pas is identified the subject is asked three questions: “Why shouldn’t they have said what they did?”; “Why do you think they did it?”; and “How do you think the person affected by the Faux-Pas feel?” In every story memory questions (control questions) are asked to ensure the subject comprehension (See supplementary material).
3. The “Empathy Quotient” Empathy allows us to understand the intentions and feelings of others and predict their behaviour. We employed this self-report questionnaire for use in adults with normal intelligence. It contains 40 empathy items and 20 fill/control items. On each empathy item, a score 2, 1 or 0 can be obtained, so the maximum score of the empathy quotient is 80.

#### Dorsolateral assessment

It was evaluated by standard tasks designed to measure executive function that depends on the dorsolateral frontal region.

1. Wisconsin card sorting test (WCST) (Heaton 1981, Psychological Assessment Resources). We employed a computerized version of this classic task measuring the ability to display flexibility in the face of changing schedules of reinforcement.
2. Letters and Numbers (WAIS-III 1997, The Psychological Corporation): This is a working memory test measuring the ability to manipulate a progressively longer piece of information.
3. Trail-making test (TMT) B: The TMT provides information on visual search, scanning, speed of processing, mental flexibility, and executive functions [18].

#### Personality

Zuckermann–Kuhlman III [19]: Self-administered 99-item questionnaire measuring impulsive sensation seeking, neuroticism-anxiety, aggression-hostility, activity, sociability and infrequency (these items detect subjects who responded carelessly).

#### Emotional state

Beck depression and anxiety inventories [20]. These questionnaires consist of 21, self-administered items about how the subject has been feeling in the last week. Each question has a set of at least four possible choices ranging in intensity.

### Statistical analysis

Descriptive statistics included frequencies and percentages, means, standard deviations (SD), and medians and range. Demographic characteristics and migraine information were compared between patients with chronic migraine and medication overuse, patients with episodic migraine and controls. Neuropsychological tests, and depression, anxiety and personality tests were also compared between the three groups. To compare categorical variables Chi-square and Fisher’s exact tests were used, and for continuous variables the analysis of variance or the non-parametric Kruskal–Wallis test was performed. The general lineal model was used to compare the neuropsychological tests between the three groups adjusting by age and depression, when necessary. Finally, all the neuropsychological tests were compared between the patients with positive course of their clinical status at 1 year of follow-up and those with negative course. For this comparison, the Student *t* test or the Wilcoxon test were performed. Kolmogorov–Smirnov test was employed to establish the normality of the distribution of the variables.

Effects were considered significant at a *p* value <0.05. All statistical analyses were performed using SAS for Windows statistical software, version 9.1 (SAS Institute, Inc., Carey, NC, USA).

## Results

The main characteristics of the three groups are depicted on Table 1.

**Table 1:** Demographics and migraine information for patients and controls

	Chronic migraine with drug overuse	Episodic migraine	Controls
*N* (females)	42 (39)	42 (35)	41 (30)
Age, mean (SD), years	41.21 (8.20)	36.19 (8.66)	37.12 (8.59)
Years of education	<12 years (17)	<12 years (16)	<12 years (9)
	12–16 years (16)	12–16 years (14)	12–16 years (17)
	>16 years (9)	>16 years (12)	>16 years (15)
Years with migraine	<5 years (5)	<5 years (9)	–
	5–15 years (13)	5–15 years (15)	
	>15 years (24)	>15 years (18)	
Medication overuse	NSAIDs (21), triptans (3), simple analgesics (4), combination of the above (14)	–	–
Preventive medication	Combination^a^ (15), amitriptyline (4), topiramate (4), zonisamide (1), beta-blockers (3), lamotrigine (1), flunaricine (1), SSRI (2), no treatment (11)	No treatment (26), beta-blockers (7), amitriptyline (7), flunaricine (2)	–
Follow-up at 1 year	Overuse persisted (24)	Continued without overuse (35)	–
	Ceased overuse (16)	Started overuse (3)	
	Lost to follow-up (2)	Lost to follow-up (4)	

*NSAIDs* Non-steroidal anti-inflammatory drugs, *SSRI* selective serotonin reuptake inhibitors

^a^More than one drug (valproic acid, zonisamide, topiramate, beta-blockers)

As expected, there was a female predominance in all groups (χ_(2)_^2^ = 5.75, *p* = 0.0563), and chronic migraine patients with medication overuse were older than episodic migraine patients and controls (χ_(2)_^2^ = 6.70, *p* = 0.0350). There were no significant differences in education years (χ_(4)_^2^ = 4.52, *p* = 0.3397) or in years with headache (χ_(2)_^2^ = 2.14, *p* = 0.3425) between CM and EM.

CM patients abused from NSAIDs, triptans, simple analgesics and a variable combination of the above. As mentioned before, we avoid using opioids in these patients, the reason why these drugs are not represented here.

Different preventive medications were or had been used in the two groups of migraineurs. Twenty-six EM patients were not on preventive medication due to the infrequent headaches, and 11 CM patients refused treatment due to the lack of consistent efficacy of prior preventive therapies.

### Neuropsychological examination (Table 2)

In terms of OF evaluation, the Faux-Pas test revealed significant differences, with the CM group performing worse than the EM group and controls. CM patients detected less Faux-Pas situations (χ_(2)_^2^ = 6.71, *p* = 0.0349) and detected a non-existent intentionality to a greater extent than the other two groups (χ_(2)_^2^ = 11.39, *p* = 0.0034). Other OF tests did not disclose significant differences. The adjusted models showed that depression was significantly associated with the task Reading the Mind in the Eyes, and in spite of this adjustment, there were still no significant differences among the three groups.

**Table 2:** Neuropsychological tests results

	Chronic migraine with drug overuse (*n* = 42)	Episodic migraine (*n* = 42)	Controls (*n* = 41)	*p*
Orbitofrontal tasks				
Faux-Pas				
Faux-Pas Composite Score	40 (0–59)	47 (0–60)	42 (25–59)	0.0397
Detected Faux-Pas	8 (0–10)	9 (0–10)	8 (5–10)	0.0349
Control questions	40 (38–40)	39.5 (37–40)	40 (37–40)	0.2115
Intentionality^a^	6 (0–10)	7 (0–10)	7 (3–10)	0.0034
Empathy^b^	7 (0–10)	8 (0–10)	8 (4–10)	0.0825
Reading the Mind in the Eyes	25 (18–31)	26 (14–32)	27 (16–35)	0.2035
Empathy quotient	47 (20–65)	42 (28–59)	47.5 (21–70)	0.2970
Dorsolateral tasks				
Trail Making B	74 (37–210)	68 (30–203)	64 (30–119)	0.1306
Letters and Numbers	10 (4–19)	10.5 (4–20)	13 (6–21)	0.0046
WCST				
Categories acquired	3 (0–5)	4 (1–5)	4 (0–6)	0.1020
Perseverative errors	7 (2–21)	6 (3–29)	5 (0–22)	0.1116
First category	12 (10–65)	11 (10–33)	12 (10–65)	0.0074

Values expressed as median (range)

*WCST* Wisconsin card sorting test

^a^Subjects answered the question: Why do you think someone did the Faux-Pas? The patient gives an incorrect answer when he/she detects intention in the Faux-Pas (which was designed as unintentional); the lower the score, the worse performance

^b^Subjects answered the question: How do you think the person affected by the Faux-Pas feels?

DL examination found that both groups of migraine patients performed worse than controls in the Letters and Numbers test (*F*_(2,121)_ = 5.62, *p* = 0.0046). CM patients had also significantly more difficulty in reaching the first category on the WCST (χ_(2)_^2^ = 9.82, p = 0.0074). The adjusted models showed that age was significantly associated with all the tasks, and depression only with Letters and Numbers and categories acquired on the WCST. After the corresponding adjustments, the results kept equal except for Trail Making B. We found that CM group performed significantly worse than controls (*p* = 0.0236).

### Emotional and personality measures (Table 3)

Depression scores of CM patients were significantly higher than the other two groups (χ_(2)_^2^ = 17.65, *p* = 0.0001), whereas both CM and EM showed higher anxiety scores than controls (χ_(2)_^2^ = 10.90, p = 0.0043). No significant differences were found on personality traits.

**Table 3:** Depression, anxiety, and personality test results

	Chronic migraine with drug overuse (*n* = 42)	Episodic migraine (*n* = 42)	Control (*n* = 41)	*p*
Beck Depression Inventory	13 (0–44)	6 (0–27)	5 (0–22)	0.0001
Beck Anxiety Inventory	12 (1–51)	11 (0–43)	6 (0–35)	0.0043
Zuckerman–Kuhlman personality questionnaire				
Impulsive sensation seeking	4 (0–15)	3 (0–11)	2.5 (0–11)	0.4670
Neuroticism–anxiety	8 (1–19)	9 (0–16)	5.5 (1–16)	0.0568
Aggression–hostility	7 (0–14)	7 (2–13)	7 (2–12)	0.6622
Activity	6 (1–16)	7 (1–15)	6.5 (1–14)	0.2660
Sociability	6 (0–9)	6 (0–14)	6 (0–12)	0.1825
Infrequency	1 (0–8)	1 (0–7)	2 (0–6)	0.3770

Values expressed as median (range)

### Follow-up (Table 4)

After 1 year of follow-up, six patients were lost, two in the CM group and four in the EM group (they refused to attend the appointments). Twenty-four of the CM patients were still abusing (60%) and 16 had stopped medication overuse. In the EM group, three patients had started medication overuse. Overall, 27 migraine patients (34%) showed a negative outcome, and 51 had a positive one (see definitions in methods).

**Table 4:** Outcome after 1 year of follow-up

	Positive outcome (*n* = 51)^a^	Negative outcome (*n* = 27)^b^	*p*
Orbitofrontal tasks			
Faux-Pas (FP)			
FP Composite Score	46 (0–60)	39 (0–52)	0.0054
Detected FP	9 (0–10)	8 (0–10)	0.0176
Who committed the FP	8 (0–10)	7.5 (0–10)	0.0329
Why is it a FP	8 (0–10)	7 (0–10)	0.0336
Why someone did the FP	6 (0–10)	4 (0–8)	0.0087
Intentionality	7 (0–10)	5 (0–9)	0.0134
Empathy	8 (0–10)	7 (0–10)	0.0150
Reading the Mind in the Eyes	27 (17–32)	23 (14–31)	0.0018
Empathy Quotient	45 (31–65)	44.5 (20–60)	0.0368
Dorsolateral tasks			
Trail Making B	68 (30–203)	79.5 (39–210)	0.0803
Letters and Numbers	10 (4–20)	9.5 (4–19)	0.4009
WCST			
Categories acquired	4 (0–5)	3 (0–5)	0.0584
Perseverative errors	6 (3–29)	7 (2–20)	0.4625
First category	11 (10–65)	12 (10–65)	0.0112
Beck Depression Inventory	8.5 (0–27)	15.5 (1–44)	0.0010
Beck Anxiety Inventory	10 (0–43)	17.5 (2–51)	0.0053
Zuckerman–Kuhlman personality questionnaire			
Impulsive sensation seeking	3 (0–11)	4.5 (0–15)	0.8960
Neuroticism–anxiety	8 (0–16)	11.5 (1–19)	0.0384
Aggression–hostility	6 (0–13)	7.5 (3–14)	0.2075
Activity	7 (1–16)	6 (1–14)	0.8264
Sociability	6 (0–14)	4.5 (0–9)	0.0805
Infrequency	1 (0–8)	1 (0–6)	0.4290

Values expressed as median (range)

^a^Patients with chronic headache that stopped medication overuse and patients with episodic headache who persisted without overuse

^b^Patients with chronic headache that persisted overusing medication and patients with episodic headache who converted into chronic migraine with medication overuse

We compared the baseline tasks performed by patients with negative and positive outcome. Patients with a positive outcome had performed significantly better on the three tasks measuring OF functioning with significant differences being present in all subtests of the Faux-Pas, the “Reading the Mind in the Eye” test and the Empathy Quotient.

No differences were found in tasks measuring DL function, except for one subtest of the WCST, where patients with a positive outcome performed better than those with a negative one in reaching the first category (*U* = 1268, *p* = 0.0112).

Patients with a negative evolution were significantly more depressed (*U* = 1207, *p* = 0.0010) and more anxious (*U* = 1143, *p* = 0.0053) than the positive outcome group.

Regarding personality traits, patients with a negative course scored significantly higher than those with a positive course in the neuroticism-anxiety factor (*t*_(67)_ = 2.11, *p* = 0.0384).

There were no between-group differences groups in terms of age, years of migraine or years of formal education.

## Discussion

Our results confirm the presence of OF dysfunction in CM patients with medication overuse from a neuropsychological perspective, and suggest that OF dysfunction heralds a poor prognosis in terms of medication overuse in these patients. In agreement with some clinical [9] and radiological studies [21], we also found a mild DL dysfunction in migraineurs (independent of overuse) as compared to controls. Migraine patients did also show greater anxiety scores than controls, and overusers were more depressed than the other two groups. The comorbidity of migraine with depression has led to suggest a shared pathogenesis [22].

In keeping with other studies, no differences were found in personality traits between migraine patients and controls [23]. We did not observe a relationship between OF dysfunction and the patients’ education level or years of migraine, which in some studies have been suggested as risk factors for poor prognosis [24]. Some authors have proposed that rather than migraine duration it is the frequency and severity of the attacks what determines a malfunctioning of the circuitries [25] implicated in pain processing and emotion, that include the OF region. The baseline frequency of episodic headaches showed a direct relationship with subsequent risk of chronic daily headache in another study [26].

The OF cortex is responsible for very complex and subjective emotional and cognitive functions that are difficult to evaluate. They include reversal learning [7], social skills, empathy, and understanding and utilizing social norms in the appropriate setting, among others [7]. We have employed a test, the Faux-Pas (see Appendix), that has been proven quite sensitive to frontal lobe damage, particularly of the ventromedial and orbitofrontal regions [16]. A recent meta-analysis also concluded that Faux-Pas detection is abnormal in the presence of frontal lobe damage, specifically of the above mentioned regions [14]. In contrast, evaluating the DL region relies on more conventional tests such as those employed in this study.

The follow-up of these patients was essential to confirm that OF cortex malfunctioning represented a poor outcome in terms of medication overuse. In accordance with the previous data, 7% of our EM patients developed medication overuse, and 60% of the CM patients persisted overusing medication [27]; it should be noted, however, that medication overuse fluctuated throughout the year in some patients. We observed that those patients with a negative outcome performed significantly worse at baseline than those with good outcome on the three OF tasks, i.e., patients with poor initial OF tests performance had a significantly higher risk of medication overuse, even if they were not abusing at study onset.

Our results and other studies [6] suggest that OF malfunctioning is present in the CM patients with medication overuse and predicts a negative outcome. Whether this OF dysfunction is the cause or is secondary to the abuse is an unsolved question but we suggest that this dysfunction is prior to abuse, since those EM patients with the worst baseline OF tests scores developed medication overuse.

The reason for this dysfunction is unclear. It could be speculated that as a result of a particular genetic background and gender (female), environmental factors (low socioeconomic status and education level, obesity), and headache characteristics (frequency, intensity and duration of pain) there would be a poorly developed prefrontal circuitry that predisposes these patients to medication overuse. Some genetic factors for medication overuse have also been described. Specifically, a polymorfism in the brain-derived neurotrophic factor (BDNF) gene (Val66Met) has been found as a significant independent predictor of analgesic consumption in patients with CM [28]. BDNF is involved in central pain sensitization and long-term potentiation. Also, wolframin His611Arg polymorphism influences medication overuse headache. Patients with chronic migraine and medication overuse harbouring that polymorphism showed a higher monthly medication consumption that non carriers. Wolframin (*WFS1*) is involved certain psychiatric illness and dependence behaviour [29]. Other polymorphisms in dopamine or serotonine receptors might be involved as these neurotransmitters control OF thalamo-striatal pathways implicated in impulse control. As a result of the prefrontal dysfunction, inhibitory response-control functions are impaired and aberrant enhancements in the limbic-striatal motivation system appear, so that patients with CM and medication overuse behave in a similar way as patients with different addictions, using medication even when it no longer produces rewarding effect (relief of pain) [30]. In contrast, DL dysfunction does not seem distinctive of overusers, being present in all types of migraineurs. Only the ability to arrange by categories, a subtest of the WCST, distinguished negative outcome patients, but this task can also be altered with OF dysfunction [31]. However, normal DL functioning is important in painful conditions, since this region exerts active control on pain perception by modulating corticosubcortical and corticocortical pathways [32]. The role of DL cortex in medication overuse cannot be inferred from our results.

In our study, other factors associated to poor prognosis were the levels of depression, anxiety, and neuroticism traits. Some authors have reported that individual differences in fear and anxiety modulate the pain response and may even cause more suffering [33]. This modulation is exerted by prefrontal mesial and orbitofrontal systems. Impulsivity trait represents a comorbidity that contributes to substance abuse although this specific trait was not overrepresented in our CM group.

This study has some limitations. First, patients with CM were older than EM patients, as reported in epidemiological studies, and age could represent a confounding factor. We performed a lineal model adjusting by age, and found no differences in our results at one-year follow-up. Second, preventive medication varied among the patients in the CM group. While 11 patients with chronic migraine were not taking any preventive medication because all drugs had failed, other patients were taking two or more (Table 1). We do not believe, however, that preventive medication played a role in their performance on the neuropsychological evaluation. Third, the levels of anxiety and depression were assessed using a self-administered psychometric tool, the Beck anxiety and depression inventory. A complete psychiatric assessment could have detected more precisely the emotional state of the patients, but was out of the scope of this study.

Based on our results, we conclude that OF dysfunction can be detected in CM patients with medication overuse with appropriate neuropsychological tests. The impairment in OF tasks predicts a poor prognosis regarding headache outcome and medication overuse in these patients. They would benefit of a neuropsychological examination with emphasis on detecting OF dysfunction, anxiety and depression so that they could benefit of an early and aggressive multidisciplinary therapeutic intervention, such as psychological and psychiatric attention, monthly neurological interviews for treatment follow-up or hospital admissions for analgesic cessation.

## Electronic supplementary material

Below is the link to the electronic supplementary material.

Supplementary material 1 (DOC 31 kb)
