# Supplementary material for: Orbitofrontal dysfunction predicts poor prognosis in chronic migraine with medication overuse
Source: J Headache Pain. 2011 Apr 17;12(4):459–66. doi: 10.1007/s10194-011-0340-6 (PMC3139058; doi:10.1007/s10194-011-0340-6)
Supplement: Supplementary file 1 — Supplementary material 1 (DOC 31 kb) [file 10194_2011_340_MOESM1_ESM.doc]

**Title**: Orbitofrontal Dysfunction Predicts Poor Prognosis In Chronic Migraine And Medication Overuse

**Journal**: Journal of Headache and Pain

**Authors**: Marian Gómez-Beldarrain, MD (corresponding author)

María Carrasco, Psych D

Amaia Bilbao, Ph D*

Juan C. García-Moncó, MD

Service of Neurology, Hospital de Galdacano

48960 Galdacano, Vizcaya, Spain

and *Fundación Vasca de Innovación e Investigación Sanitarias (BIOEF)
48150  Sondika (Bizkaia)

Corresponding author: mgomezab@sarenet.es

**SUPPLEMENTARY MATERIAL**

**Example of Faux-Pas story:**

Jean West, a manager in Abco Software Design, called a meeting for all of the staff. "I

have something to tell you," she said. "John Morehouse, one of our accountants, is very sick with cancer and he's in the hospital." Everyone was quiet, absorbing the news, when Robert, a software engineer, arrived late. "Hey, I heard this great joke last night!” Robert said. “What did the terminally ill patient say to his doctor?" Jean said, "Okay, let's get down to business in the meeting."

Did anyone say something they shouldn't have said or something awkward?

**If yes,** ask:

Who said something they shouldn't have said or something awkward?

Why shouldn't he/she have said it or why was it awkward?

Why do you think he/she said it?

When he came in, did Robert know that the accountant was sick with cancer?

How do you think Jean, the manager, felt?

**Control question:** In the story, what did Jean, the manager, tell the people in the meeting?

Who arrived late to the meeting?

**Example of no Faux-Pas story**

Joan took her dog, Zack, out to the park. She threw a stick for him to chase. When they

had been there a while, Pam, a neighbor of hers, passed by. They chatted for a few minutes. Then Pam asked, "Are you heading home? Would you like to walk together?" "Sure," Joan said. She called Zack, but he was busy chasing pigeons and didn't come. "It looks like he's not ready to go," she said. "I think we'll stay." "OK," Pam said. "I'll see you later."

Did anyone say something they shouldn't have said or something awkward?

**If yes,** ask:

Who said something they shouldn't have said or something awkward?

Why shouldn't he/she have said it or why was it awkward?

Why do you think he/she said it?

When she invited her, did Pam know that Joan wouldn’t be able to walk home with her?

How do you think Pam felt?

**Control question:** In the story, where had Joan taken Zack?

Why didn’t she walk with her friend Pam?

<http://www2.psy.uq.edu.au/~stone/order.html>

Reference: Baron-Cohen S, O'Riordan M, Stone V, Jones R, Plaisted K (1999) Recognition of faux pas by normally developing children and children with Asperger syndrome or high-functioning autism. *J Autism Dev Disord*. 29:407-418
